# Immunoregulatory Imbalance in Preeclampsia: A Cross-Sectional Study of B7-H3 and Decidual NK Cell Interactions

**DOI:** 10.3390/medsci13040239

**Published:** 2025-10-22

**Authors:** Khanisyah Erza Gumilar, Alexander Indra Humala, Manggala Pasca Wardhana, Ernawati Ernawati, Agus Sulistyono, Budi Utomo, Grace Ariani, Ming Tan, Erry Gumilar Dachlan, Gus Dekker

**Affiliations:** 1 Department of Obstetrics and Gynecology, Universitas Airlangga Hospital, Surabaya 60115, Indonesia; 2 Department of Obstetrics and Gynecology, Faculty of Medicine, Universitas Airlangga, Surabaya 60132, Indonesia; alexihs.og@gmail.com (A.I.H.); manggala.pasca@fk.unair.ac.id (M.P.W.); ernawati@fk.unair.ac.id (E.E.); agussulsby@yahoo.com (A.S.); mingtan@cmu.edu.tw (M.T.); errygumilar@gmail.com (E.G.D.); gustaaf.dekker@adelaide.edu.au (G.D.); 3 Department of Obstetrics and Gynecology, Indonesia Army Central Hospital Gatot Soebroto, Jakarta 10410, Indonesia; 4 Department of Obstetrics and Gynecology, Dr. Soetomo General Academic Hospital, Surabaya 60286, Indonesia; 5 Department of Public Health and Preventive Medicine, Faculty of Medicine, Universitas Airlangga, Surabaya 60132, Indonesia; budiutomo@fk.unair.ac.id; 6 Department of Pathology Anatomy, Universitas Airlangga, Surabaya 60132, Indonesia; graceariani_dr@yahoo.com; 7 Graduate Institute of Biomedical Science, China Medical University, Taichung 40402, Taiwan; 8 Institute of Biochemistry and Molecular Biology and Research Center for Cancer Biology, China Medical University, Taichung 406040, Taiwan; 9 Department of Obstetrics and Gynecology, Lyell McEwin Hospital, University of Adelaide, Adelaide, SA 5005, Australia

**Keywords:** preeclampsia, B7-H3, dNK cells, Immunotolerance, maternal–fetal interface

## Abstract

Background: Preeclampsia remains a leading cause of maternal and perinatal morbidity and mortality, yet its pathophysiology is not fully understood. Recent studies suggest that dysregulated maternal immune responses, particularly involving decidual Natural Killer (dNK) cells and immune checkpoint molecules such as B7-H3, may play a role in the pathogenesis of this heterogeneous syndrome, particularly in the development of early-onset preeclampsia (EOP). Objective: The aim of this study was to investigate the expression patterns of B7-H3 on extravillous trophoblasts (EVTs) and the abundance of dNK cells in preeclamptic versus normotensive pregnancies and to analyze the relationship between these two immune parameters. Methods: A cross-sectional study was conducted using 42 placental samples (21 preeclampsia, 21 controls). Immunohistochemistry (IHC) was performed to detect CD56 (dNK cells) and CD276 (B7-H3) expression. Expression was semi-quantitatively evaluated using the Remmele Immunoreactive Score (IRS). Statistical comparisons and correlation analyses were conducted. Results: Preeclamptic placentas exhibited significantly higher dNK cell expression (IRS 7.19 ± 2.16) and significantly lower B7-H3 expression (IRS 2.63 ± 0.90) compared to controls (*p* < 0.001 and *p* = 0.002, respectively). A positive correlation was found between B7-H3 and dNK cell expression in both groups, with a stronger correlation in normotensive pregnancies (r = 0.605; *p* = 0.004) and preeclampsia (r = 0.465; *p* = 0.034). Conclusions: The inverse expression pattern and reduction in B7-H3 expression compared to dNK cells in preeclampsia suggest a loss of immune tolerance at the maternal–fetal interface. These findings highlight the potential of B7-H3 as a biomarker and immunoregulatory target for early prediction and prevention of preeclampsia.

## 1. Introduction

Preeclampsia is a multisystem pregnancy disorder characterized by new-onset hypertension after 20 weeks of gestation, accompanied by complications such as proteinuria or maternal/uteroplacental end-organ dysfunction [1]. It remains one of the leading causes of maternal and fetal morbidity and mortality worldwide, with an estimated global incidence of 2–8% of all pregnancies [2]. The pathophysiology of preeclampsia is highly complex, with several proposed mechanisms, including placental dysfunction [3,4], oxidative stress [5,6], ferroptosis [7,8], and maternal immune maladaptation [9]. Preeclampsia is a heterogeneous syndrome with distinct pathophysiological mechanisms. Early-onset preeclampsia (EOP), resulting in iatrogenic preterm birth before 34 weeks, is commonly linked to placental dysfunction and fetal growth restriction due to inadequate cytotrophoblast invasion of spiral arteries [9]. In contrast, late-onset preeclampsia (LOP), particularly at term (>37 weeks), is more often associated with maternal factors such as obesity, inflammation, and advanced maternal age, reflecting a pathogenesis driven by premature placental senescence rather than impaired trophoblast invasion.

A failure of maternal immune tolerance toward trophoblast invasion during early pregnancy is believed to play a central role in the etiology of EOP. A normal maternal immune response to trophoblasts involves a delicate balance among decidual immune cells, including dNK, macrophages, T cells, and dendritic cells [10,11]. Proper immune tolerance is essential for successful embryo implantation and placental development. In contrast, immune maladaptation can lead to defective placentation, followed by oxidative stress and placental inflammation, ultimately resulting in the clinical manifestations of EOP [12].

dNK cells are the dominant immune cell population in the uterine decidua during early pregnancy, playing a crucial role in implantation and the formation of a healthy placenta. dNK cells exhibit low cytotoxicity and instead promote pregnancy by producing ‘type 2’ cytokines and angiogenic factors that facilitate trophoblast invasion and remodeling of the uterine spiral arteries [13,14]. The immune interaction between dNK cells and trophoblasts is mediated in part by the expression of non-classical class I Human Leukocyte Antigens (HLA), such as HLA-C, on trophoblasts, which are recognized by Killer Immunoglobulin Receptors (KIRs) on dNK cells [15]. Disruption in the interaction between dNK cells and trophoblasts, particularly due to the maternal KIR AA genotype lacking activating receptors, may impair dNK-mediated modulation of trophoblast invasion. This imbalance contributes to defective placentation [16,17]. In addition to the KIR/HLA interaction, trophoblasts also express immunomodulatory molecules from the B7 family that regulate their interaction with maternal immune cells [18]. One such molecule is B7-H3 (also known as CD276), an adaptive immune co-stimulatory protein. B7-H3 is consistently expressed in the placenta from the first trimester through term, particularly on EVT cells [19]. In pregnancy, B7-H3 is involved in regulating adaptive immune responses through the activation or inhibition of lymphocytes, including NK and T cells [20]. Specifically, B7-H3 has been shown to influence dNK cell function by modulating the secretion of pro-inflammatory cytokines by dNK cells. Emerging evidence suggests that reduced expression of B7-H3 is associated with increased production of pro-inflammatory cytokines by dNK cells, as well as impaired trophoblast migration and invasion [21]. In cases of recurrent miscarriage, low B7-H3 expression on trophoblasts has been reported to trigger excessive inflammatory cytokine secretion by dNK cells and inhibit trophoblast invasion [21], potentially resulting in inadequate placentation and in theory, a clinical condition resembling EOP if pregnancy continues.

Despite growing insights into the role of B7-H3 in regulating immune tolerance during pregnancy, studies specifically examining its expression and function in the context of preeclampsia remain scarce. To date, no previous research has directly evaluated the relationship between B7-H3 expression on trophoblasts and the presence or activity of dNK in preeclampsia. Therefore, this study was conducted to investigate the differences in B7-H3 expression on EVT and the number of decidual NK cells between preeclamptic and normal pregnancies and to analyze the relationship between the two. The findings of this study are expected to deepen our understanding of the immunological mechanisms underlying preeclampsia. They may provide a foundation for the development of novel therapeutic strategies, such as B7-H3 modulation, to prevent preeclampsia in the future.

## 2. Materials and Methods

### 2.1. Design and Subjects

This study employed an analytical observational research design with a cross-sectional approach. A total of 42 placental samples were collected post-delivery and divided into two groups: 21 samples from pregnancies with preeclampsia (based on the criteria of the International Society for the Study of Hypertension in Pregnancy/ISSHP) [22] and 21 samples from normotensive pregnancies as controls.

Inclusion criteria comprised intrauterine singleton pregnancy, maternal age ≥ 18 years, informed consent to participate, and fulfillment of the diagnostic criteria for either preeclampsia or normal pregnancy. Exclusion criteria included chronic hypertension, gestational hypertension without preeclampsia, diabetes mellitus, autoimmune diseases, placental abnormalities (e.g., hydatidiform mole), or other severe maternal complications. The study subjects were pregnant women who delivered at Dr. Soetomo General Academic Hospital (ethical clearance No. 1014/KEPK/VI/14 June 2024) and Universitas Airlangga Hospital (ethical approval No. 063/KEP/27 April 2024), Surabaya, Indonesia.

### 2.2. Sample Collection Procedure

The decidua basalis tissue of the placenta was immediately fixed in buffered formalin and processed into paraffin blocks. Tissue sections with a thickness of 4 µm were prepared for immunohistochemical staining. Two markers were examined on serial sections: the dNK cell marker using monoclonal anti-CD56 antibody, and the B7-H3 marker on trophoblasts using monoclonal anti-CD276 antibody. The streptavidin-biotin immunohistochemistry method was applied according to standard protocols.

Briefly, after deparaffinization and rehydration, antigen retrieval was performed using heat-induced epitope retrieval, followed by blocking of endogenous peroxidase activity. The sections were then incubated overnight at 4 °C. IHC staining was performed using the CD56 antibody (BC56C04, Biocare Medical, Pacheco, CA, USA) and the monoclonal anti-CD276 antibody (Invitrogen MA515693, Thermo Fisher Scientific, Waltham, MA, USA).

The next day, the slides were washed with PBS and incubated with a biotin-labeled secondary antibody, followed by the addition of streptavidin-HRP. The antigen–antibody complexes were visualized using diaminobenzidine, which produced a brown coloration in positively stained cells. Negative controls were performed using identical tissue sections that were processed without the addition of primary antibodies.

### 2.3. Expression Assessment

The stained slides were examined under the microscope (Nikon^®^ Eclipse CI light). For each specimen, five random fields within the decidua basalis area containing EVT were selected, confirmed at 40× magnification, and subsequently analyzed at 200× magnification. The expression of dNK cells and B7-H3 was evaluated by identifying brown chromogenic staining in the target cells.

**Figure 1:**
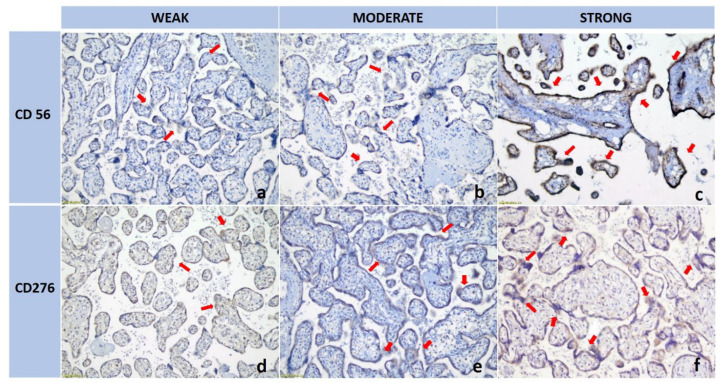
dNK (CD56; top) and B7-H3 (CD276; bottom) Expression (indicated by arrows): (**a**) weak dNK expression; (**b**) moderate dNK expression; (**c**) strong dNK expression; (**d**) weak B7-H3 expression; (**e**) moderate B7-H3 expression; (**f**) strong B7-H3 expression. 200× magnification by Nicon Eclipse Ci series.

CD56-positive dNK cells were identified as large granular cells exhibiting brown membranous or cytoplasmic staining within the decidual stroma surrounding the trophoblasts. B7-H3 expression in EVT appeared as brown staining localized to the membranes and cytoplasm (Figure 1). Quantification was performed semi-quantitatively using the Remmele Immunoreactive Score (IRS). This composite score combines the percentage of immunoreactive cells and the staining intensity (Remmele & Stegner, with methodological modifications) [23,24]. The mean IRS for each marker was calculated from five fields per sample, resulting in average expression values for dNK and B7-H3. IRS evaluation was performed independently by two blinded observers. In cases where scoring discrepancies occurred, the slides were re-examined, and consensus was achieved through joint review to ensure consistency and reproducibility.

CD56 expression is primarily localized to specific cell populations in the decidual stroma, as indicated by red arrows, with variations in distribution patterns observed across the panels (Figure 1a–c). In the B7-H3 series, a progressive increase in staining intensity is evident from panel Figure 1d–f, particularly along the membranes and within the cytoplasm of trophoblastic cells (also marked by red arrows), suggesting an upregulation of B7-H3 expression. This comparative analysis underscores the distinct biological roles of CD56 and B7-H3 in modulating immune responses and contributing to trophoblast differentiation within the placental microenvironment.

### 2.4. Data Analysis

Numerical data were expressed as mean ± standard deviation. An unpaired *t*-test was used to compare the mean expression levels of CD56 (dNK cells) and B7-H3 (CD276) between the preeclampsia and control groups, as both variables were normally distributed within each group according to the Shapiro–Wilk test. In addition, Pearson or Spearman correlation analysis was conducted to assess the relationship between B7-H3 expression and the number of dNK cells in both the preeclampsia and control groups, depending on data distribution. A *p*-value of <0.05 was considered statistically significant. All statistical analyses and data visualizations were carried out using GraphPad Prism version 8.2.1 (GraphPad Software, San Diego, CA, USA).

## 3. Results

A total of 42 placental samples were analyzed (21 from the preeclampsia group and 21 from the control group). There were no significant differences in maternal age, parity, or BMI between the groups (*p* > 0.05). A significant difference was observed in neonatal birth weight (*p* < 0.05) (Table 1).

### 3.1. dNK Cell Expression Was Higher in the Preeclamptic Placenta than in the Normotensive Placenta

**Figure 2:**
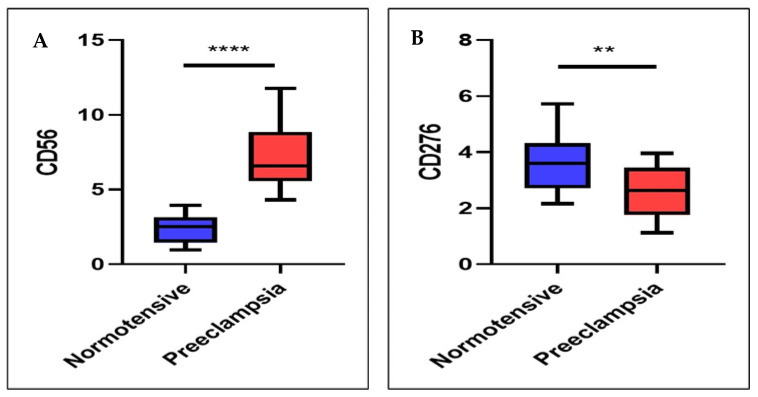
The expression levels of CD56 and CD276 between the two groups (Normotensive vs. preeclampsia). (**A**) CD56 expression is significantly higher in the Preeclampsia group compared to the Normotensive group. (**B**) CD276 expression is significantly lower in the Preeclampsia group than in the Normotensive group. ** = *p*-value less than 0.01; **** = *p*-value less than 0.0001.

IHC analysis demonstrated the presence of dNK cells dispersed within the decidual stroma surrounding the trophoblasts. Quantitatively, dNK cell expression (reflecting the number of dNK cells) differed significantly between the preeclampsia and control groups. The mean dNK cell IRS in preeclamptic placentas was 7.19 ± 2.16. significantly higher than in normotensive pregnancies. which was 2.42 ± 0.96 (*p* < 0.001) (Table 2; Figure 2A). These findings indicate an increased number of dNK cells in preeclamptic conditions.

### 3.2. B7H3 Expression Was Lower in the Preeclamptic Placenta than in the Normotensive Placenta

B7-H3 expression was observed on the membrane/cytoplasm of EVT within decidual tissue. The B7-H3 expression average in the preeclampsia group was 2.63 ± 0.90, significantly lower than that in the control group, which was 3.62 ± 0.99 (*p* = 0.002) (Table 2; Figure 2B). These findings suggest that trophoblasts in preeclamptic pregnancies exhibit reduced expression of the B7-H3 molecule compared to those in normal pregnancies.

### 3.3. The Relationship Between B7-H3 Expression and dNK Cells

**Figure 3:**
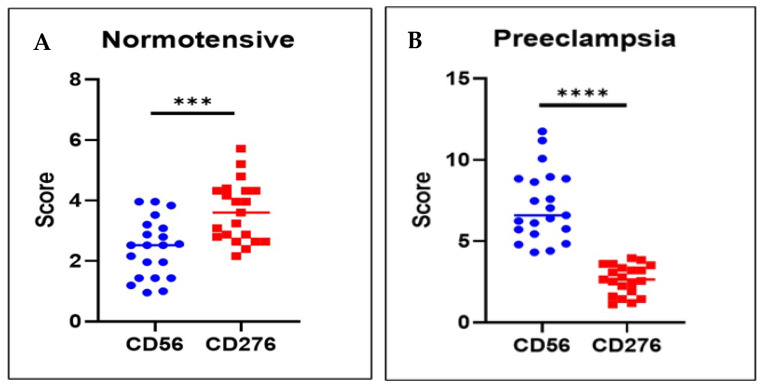
Correlation of dNK cell and B7H3 in the two groups. (**A**) In normotensive pregnancies, the expression of CD276 (B7-H3) is more prominent than that of CD56, which reflects a balanced immune-modulating environment at the maternal–fetal interface, potentially facilitating adequate immune tolerance and regulation of dNK cells. (**B**) In preeclampsia, there is a marked increase in CD56, suggesting an elevated number or activity of dNK cells, which may indicate an inflammatory or maladaptive immune response. Conversely, the reduced CD276 expression suggests a decrease in immune tolerance or checkpoint signaling, which could contribute to the pathophysiology of preeclampsia by allowing excessive immune activation and impaired trophoblast invasion. *** = *p*-value less than 0.001; **** = *p*-value less than 0.0001.

In normotensive pregnancies, the expression of dNK cells differs significantly from that of B7-H3, with it showing markedly higher expression levels (Figure 3A). In contrast, in preeclamptic pregnancies, dNK cell expression is considerably higher than B7-H3 expression (*p* < 0.001) (Figure 3B).

Correlation analysis demonstrated a significant positive association between B7-H3 expression in trophoblasts and the number of dNK cells in both normotension and preeclampsia. When analyzed within each group separately, a significant positive correlation was observed between B7-H3 expression and dNK cell abundance in both groups: normotensive (r = 0.605; *p* < 0.01) and preeclampsia (r = 0.465; *p* < 0.05) (Table 3). This indicates that, under each condition, placentas with relatively higher B7-H3 expression also tended to have more dNK cells. However, when comparing the groups, preeclampsia was characterized by an overall pattern of decreased B7-H3 expression alongside increased abundance of dNK cells, reflecting a disease-specific inverse trend.

**Figure 4:**
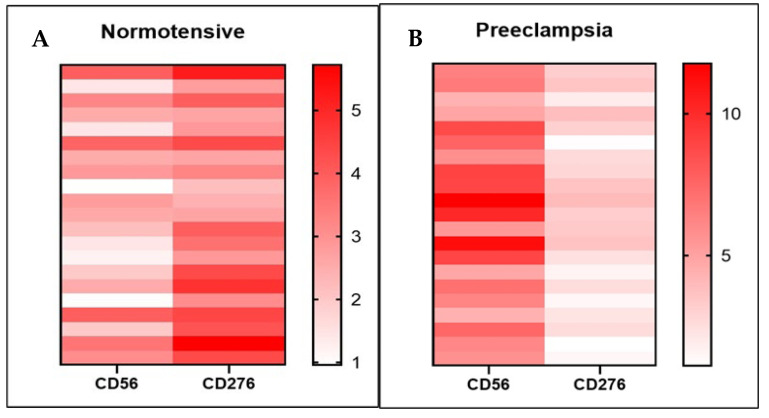
Heatmap visualization of B7-H3 and dNK expression in both groups. The normotensive pregnancy group (**A**) exhibits higher expression of B7-H3 (CD276) accompanied by relatively low expression of decidual natural killer (dNK) cells (CD56). In contrast, preeclampsia (**B**) is characterized by an increased abundance of dNK and reduced expression of B7-H3.

A detailed examination within each group reveals notable inter-sample variability in expression patterns. Through heatmap visualization (Figure 4A,B), distinct differences in the expression levels of dNK cells and B7-H3 across individual samples become evident. These findings provide further support for a strong inverse association between B7-H3 expression and dNK cell abundance, whereby lower B7-H3 levels are consistently associated with elevated dNK cell expression and vice versa.

## 4. Discussion

This pilot case–control study demonstrates significant alterations in maternal–fetal immune components in preeclampsia, characterized by decreased expression of B7-H3 on EVT and an increased number of dNK cells. B7-H3, a member of the B7 family of immune checkpoint molecules, plays a complex and context-dependent role in immunoregulation during pregnancy. B7-H3 was initially identified as a co-stimulatory molecule that enhances CD4^+^/CD8^+^ T cell proliferation and IFN-γ production. However, subsequent studies have reported opposing findings suggesting that B7-H3 may also function as a negative immune checkpoint. Thus, its role in T cell regulation appears to be context-dependent and remains a subject of ongoing controversy [25]. B7-H3 mRNA expression is markedly elevated in placental tissue compared to other normal tissues [26], strongly suggesting its role as a “tolerogenic molecule” that may contribute to the maintenance of maternal immune tolerance toward the fetus.

During normal gestation, maternal immune tolerance is crucial for maintaining fetal survival, and this process involves intricate interactions between decidual immune cells and trophoblasts. B7-H3 is expressed at the maternal–fetal interface, particularly by EVT and stromal cells, where it is believed to contribute to immune tolerance by modulating the activity of dNK cells and other maternal immune cells [19,27]. Its immunosuppressive properties may help prevent an excessive maternal immune response against the semi-allogeneic fetus, facilitating successful implantation, placental development, and fetal growth.

Conversely, dysregulated expression of B7-H3 has been implicated in the pathogenesis of disorders characterized by impaired placentation. Emerging evidence suggests that alterations in B7-H3 expression are involved in the underlying pathophysiology of unexplained recurrent pregnancy loss [21]. Reduced B7-H3 expression is associated with heightened production of pro-inflammatory cytokines by dNK cells, which may exacerbate immune dysregulation and compromise trophoblast invasion and vascular remodeling during early pregnancy, ultimately contributing to pregnancy failure.

The preeclampsia cases in this study demonstrate decreased expression of B7-H3 on EVT and an increased number of dNK cells. In normal pregnancy, high B7-H3 expression on trophoblasts appears to correlate positively with a well-regulated. This reflects a state of immune tolerance, wherein trophoblasts and maternal immune cells interact harmoniously. B7-H3 functions as an immunoregulatory molecule that modulates the maternal immune response to the fetus, allowing for the presence of a substantial number of dNK cells with a tolerogenic, non-cytotoxic phenotype.

A positive correlation between B7-H3 expression and dNK cell abundance suggests that a strongly immunotolerant decidual environment permits a greater presence of dNK cells without triggering maternal immune rejection of the fetus. In healthy pregnancy, dNK cell populations undergo dynamic changes across gestation. They are highly abundant during the first trimester, comprising roughly 50–70% of decidual lymphoid cells [28,29]. As pregnancy progresses into the second and third trimesters, the number and proportion of dNK cells in the decidua gradually decline [13].

These findings may appear paradoxical at first glance; however, they represent two complementary levels of analysis. The positive correlation observed within each group reflects inter-sample variability, suggesting that B7-H3 may still exert an immunoregulatory influence on dNK cells regardless of clinical status. On the other hand, the overall inverse pattern observed between normotensive and preeclamptic groups highlights a disease-related shift in baseline expression, lower B7-H3 levels accompanied by higher dNK abundance in preeclampsia. Thus, the apparent discrepancy can be reconciled by distinguishing between within-group associations and between-group differences, both of which underscore the contribution of B7-H3/dNK imbalance to the pathophysiology of preeclampsia.

In contrast, preeclampsia is characterized by significantly reduced B7-H3 expression accompanied by an increased accumulation of dNK cells. This downregulation of B7-H3 suggests a partial loss of immunoinhibitory signaling in the preeclamptic placenta, potentially leading to dysregulation of maternal immune cell activity. The elevated number of dNK cells could reflect an exaggerated immune response or a compensatory reaction to underlying placental stress.

It is well recognized that preeclampsia involves substantial placental stress even at term. Similarly, in term preeclampsia, a dysfunctional syncytiotrophoblast (STB) releases pro-inflammatory and anti-angiogenic factors that drive the maternal syndrome [12]. One consequence of this stress is activation of the complement system in the placental microenvironment. Complement overactivation triggers an inflammatory cascade, generating anaphylatoxins that recruit and activate immune cells [30,31]. These processes create a pro-inflammatory milieu in the preeclamptic placenta, providing a mechanistic context for the observed increase in dNK cell accumulation when B7-H3 is downregulated, thereby linking placental stress with innate immune cell recruitment in preeclampsia.

Activated dNK cells can secrete pro-inflammatory cytokines, which may hinder trophoblast invasion and disrupt uterine artery remodelling. Thus, our findings support the hypothesis of immune maladaptation in preeclampsia, where diminished expression of tolerogenic molecules, such as B7-H3, leads to uncontrolled effector immune responses, ultimately contributing to shallow placentation and maternal endothelial dysfunction.

In our study, only 2 out of 21 preeclamptic pregnancies were complicated by fetal growth restriction (FGR). This underrepresentation likely reflects the clinical case distribution in our hospitals during the study period rather than a true absence of the phenotype. While this limits the power to explore the immunological alterations specific to severe preeclampsia with FGR, our findings still highlight a consistent pattern of B7-H3 downregulation and dNK cell increase across preeclamptic cases.

The majority of preeclamptic pregnancies are born after 34 weeks, with most neonates weighing more than 2000 g. This pattern is consistent with the prevalence of late-onset preeclampsia in our data, which is less related to reduced fetal growth than early-onset illness. These findings highlight the variability of preeclampsia and demonstrate that immunological changes at the maternal–fetal interface can occur even in the absence of significant fetal impairment. This is notable because preeclampsia with FGR is often linked to shallow EVT invasion of the uterine wall, resulting in inadequate spiral artery remodeling and consequent uteroplacental insufficiency [4,32]. The presence of FGR in these placentas likely indicates deficient trophoblast invasion [33], reinforcing our interpretation that reduced B7-H3 expression and subsequent immune dysregulation contribute to shallow EVT invasion and poor placental perfusion. Overall, our findings align with established mechanisms of shallow placentation in preeclampsia, and the observed interplay of STB stress, complement-driven inflammation (with decidual NK cell recruitment) [34,35], and FGR further supports the role of shallow trophoblast invasion in such cases [36].

It should also be emphasized that the majority of our preeclamptic cohort consisted of late-onset cases, with only two pregnancies complicated by FGR. This distribution likely explains the relatively preserved gestational age at delivery and the lower incidence of severe neonatal complications in our study. While adverse fetal outcomes are more typically associated with the early-onset preeclampsia phenotype accompanied by FGR, our findings demonstrate that downregulation of B7-H3 and expansion of dNK cells were consistently present across preeclampsia cases, regardless of FGR status. This suggests that immune maladaptation at the maternal–fetal interface may represent a broader pathogenic mechanism in preeclampsia, not limited to those cases with severe fetal compromise. Nevertheless, the relatively mild fetal phenotype in our series should be taken into account when interpreting the clinical implications of these immunological alterations.

This study is also in tone with previous reports highlighting the critical role of immune checkpoint molecules in the placenta [21]. Reduced B7-H3 expression on trophoblasts has been associated with recurrent pregnancy loss, potentially through mechanisms involving increased cytokine secretion by NK cells that impair trophoblast invasion. Although the clinical contexts differ (miscarriage versus preeclampsia), both conditions reflect a common underlying scenario: the deficiency of immunoregulatory signals leads to a more pro-inflammatory decidual environment that is less conducive to successful placentation.

Our findings provide additional evidence that immune regulation at the maternal–fetal interface involves not only the HLA system but also the B7-H3/CD276 pathway. It is important to note that the increased number of dNK cells observed in preeclamptic placentas does not necessarily indicate normal functional activity. It is plausible that the dNK cells in preeclampsia represent a subpopulation with a more cytotoxic or pro-inflammatory phenotype compared to those found in normal pregnancy.

The increased number of dNK cells may also result from the recruitment of immune cells in response to placental hypoxia or tissue damage, suggesting that their accumulation could be a consequence of preeclampsia development [30,37]. Additionally, the cross-sectional design limits our ability to establish a causal relationship between B7-H3 downregulation and the development of preeclampsia. The observed associations should therefore be interpreted as preliminary, and future prospective studies are warranted to clarify whether reduced B7-H3 expression precedes and contributes to disease onset. Although the exact cause-and-effect relationship is unclear, the moderate but significant correlation in the preeclamptic group suggests that higher B7-H3 expression is generally linked to a greater number of dNK cells, even under disease conditions.

### 4.1. Limitations of the Study

This study has several limitations. First, the cross-sectional design at the time of delivery may not fully capture the dynamic changes in the placenta during early pregnancy. However, the consistent expression of B7-H3 on EVT in late gestation is in line with previous studies. Then, the relatively small sample size limits the statistical power and generalizability of the results. This pilot study likely reflects population-level trends, but the findings may vary when stratified by preeclampsia phenotypes. Notably, the classical EOP phenotype associated with FGR was underrepresented in our cohort. Even when applying a threshold of the 25th percentile for birthweight, only 4 out of 21 preeclamptic cases met this criterion. This scarcity of classical EOP with FGR should be acknowledged as a key limitation of our study. Furthermore, the limited representation of severe preeclampsia with FGR in our cohort restricts the ability to evaluate the impact of immune imbalance on fetal outcomes directly. As most of our cases were late-onset preeclampsia with relatively preserved fetal growth, the translational significance of the observed immunological alterations may not fully capture the spectrum of disease severity. Future research involving larger, phenotype-specific cohorts is necessary to enable a more robust comparison between different preeclampsia subtypes, including normotensive FGR, and to validate the immunological patterns observed in this preliminary investigation.

### 4.2. Future Perspective

From a clinical perspective, our findings highlight the potential of B7-H3 and dNK cells as future biomarkers and therapeutic targets in preeclampsia. If reduced B7-H3 expression can be reliably detected early in pregnancy, this molecule could be developed as part of a predictive risk assessment tool for preeclampsia, complementing existing screening models. Similarly, interventions aimed at enhancing immunotolerant signalling at the placental interface, either by enhancing B7-H3 signaling or modulating excessive dNK activation, represent potential avenues for innovative therapeutic development in obstetrics.

Based on a screening perspective, if validated in larger longitudinal cohorts, B7-H3 could be integrated into existing first-trimester risk assessment models, which currently combine maternal history, blood pressure, uterine artery Doppler, and angiogenic biomarkers (e.g., PlGF, sFlt-1). As an immunological marker, B7-H3 may provide complementary insights into maternal–fetal immune tolerance, potentially improving predictive accuracy for early detection of high-risk pregnancies.

From a therapeutic perspective, approaches that strengthen B7-H3 signaling or temper excessive dNK activation may provide novel options for managing preeclampsia, although their application remains at an early and investigational stage. These strategies align with the emerging paradigm of immunotherapy in obstetrics, though feasibility, safety, and efficacy must be carefully established through preclinical studies and phased clinical trials before clinical application. To advance these concepts, longitudinal studies beginning in early pregnancy are essential. Such research should incorporate serial maternal blood sampling, placental tissue analysis, and advanced methodologies, including single-cell RNA sequencing and spatial transcriptomics, to map temporal immune dynamics and clarify causal pathways.

Ultimately, multi-center validation studies will be required to confirm B7-H3′s predictive value and to translate immunoregulatory modulation into safe, effective interventions for women at high risk of preeclampsia.

## 5. Conclusions

This study provides compelling evidence of a disrupted immunological environment in preeclamptic placentas, characterized by reduced expression of the immune checkpoint molecule B7-H3 and a concomitant increase in dNK cell numbers. The strong correlation between B7-H3 and dNK cells underscores the critical role of immune regulation at the maternal–fetal interface. These findings support the hypothesis that impaired immune tolerance contributes to preeclampsia pathogenesis. B7-H3 holds promise as a novel biomarker and potential therapeutic target in preeclampsia. Future longitudinal studies are warranted to explore the predictive value of B7-H3 in early pregnancy and to evaluate strategies aimed at restoring immune balance in high-risk pregnancies.

## Figures and Tables

**Table 1:** Sample characteristics.

Item	Preeclampsia (n = 21)	Normotensive (n = 21)	*p*-Value
Maternal Age (years)			
≤30	9 (42.9%)	13 (61.9%)	0.354
>30	12 (57.1%)	8 (38.1%)	
Gestational Age (weeks)			
<37	11 (52.4%)	2 (9.5%)	0.006
≥37	10 (47.6%)	19 (90.5%)	
Parity			
Primigravida	5 (23.8%)	6 (28.6%)	1.000
Multigravida	16 (76.2%)	15 (71.4%)	
BMI			
Normal	3 (14.3%)	9 (42.9%)	0.160
Overweight	8 (38.1%)	5 (23.8%)	
Obesity I	5 (23.8%)	6 (28.6%)	
Obesity II	4 (19.0%)	1 (4.8%)	
Obesity III	1 (4.8%)	0 (0.0%)	
Birth weight (grams)			
<2000	7 (33.3%)	0 (0.0%)	0.009
≥2000	14 (66.7%)	21 (100%)	
Percentile of birthweight			
75–90	7 (33.3%)	5 (23.8%)	0.453
50–75	4 (19.0%)	8 (38.1%)	
25–50	6 (28.6%)	6 (28.6%)	
10–25	2 (9.5%)	2 (9.5%)	
<10	2 (9.5%)	0 (0.0%)	
Type of Preeclampsia (n = 21)			
EOP (<34 weeks)	6 (28.6%)		
LOP (≥34 weeks)	15 (71.4%)		
Gestational Age in Preeclampsia (weeks) (n = 21)			
<28	2 (9.5%)		
28–<34	4 (19.1%)		
≥34	15 (71.4%)		

BMI: Body mass index; EOP; Early-onset preeclampsia; LOP: Late-onset preeclampsia.

**Table 2:** Mean Expression of dNK Cells (CD56) and B7-H3 (CD276).

Marker	Mean Expression (IRS)—Preeclampsia (n = 21)	Mean Expression (IRS)—Normotensive (n = 21)	*p*-Value
dNK (CD56)	7.19 ± 2.16	2.42 ± 0.96	<0.001 ^1^
B7-H3 (CD276)	2.63 ± 0.90	3.62 ± 0.99	0.002 ^1^

^1^ Independent *t*-test; significant difference (*p* < 0.05).

**Table 3:** Correlation between B7-H3 and dNK cells in both groups.

Subject	n	*p*-Value	r (Correlation Coefficient)
Normotensive	21	0.004	0.605
Preeclampsia	21	0.034	0.465

## Data Availability

The data presented in this study are available on request from the corresponding author. The data are not publicly available due to privacy or ethical restrictions.
